# Availability of and factors related to interventional procedures for refractory pain in patients with cancer: a nationwide survey

**DOI:** 10.1186/s12904-022-01056-6

**Published:** 2022-09-26

**Authors:** Yuko Uehara, Yoshihisa Matsumoto, Toshifumi Kosugi, Miyuki Sone, Naoki Nakamura, Akio Mizushima, Mitsunori Miyashita, Tatsuya Morita, Takuhiro Yamaguchi, Eriko Satomi

**Affiliations:** 1 Department of Palliative Medicine, Juntendo University Graduate School of Medicine, 2-1-1 Hongo, Bunkyo-ku, Tokyo, 113-8421 Japan; 2 Department of Palliative Medicine, National Cancer Center Hospital East, Kashiwa, Japan; 6-5-1 Kashiwanoha, Kashiwa, Chiba, 277-8577 Japan; 3 Department of Palliative Therapy, Cancer Institute Hospital of Japanese Foundation for Cancer Research, Tokyo, Japan; 3-8-31 Ariake, Koto-ku, Tokyo, 135-8550 Japan; 4 Department of Palliative Care, Saga-ken Medical Centre Koseikan, Saga, Japan; 400 Kasemachinakabaru, Saga, Saga 840-8571 Japan; 5 Department of Diagnostic Radiology/Interventional Radiology Center, National Cancer Center Hospital, Tokyo, Japan; 5-1-1 Tsukiji, Chuo-ku, Tokyo, 104-0045 Japan; 6 Department of Radiology, St. Marianna University School of Medicine, Kawasaki, Japan; 2-16-1 Sugao, Miyamae-ku, Kawasaki, Kanagawa 216-8511 Japan; 7 Department of Palliative Nursing, Health Sciences, Tohoku University Graduate School of Medicine, Sendai, Japan; 2-1 Seiryo-machi, Aoba-ku, Sendai, Miyagi 980-8575 Japan; 8 Division of Supportive and Palliative Care, Seirei Mikatahara General Hospital, Hamamatsu, Japan; 3453 Mikatahara-cho, Hamamatsu, Shizuoka, 433-8558 Japan; 9 Division of Biostatistics, Tohoku University School of Medicine, Sendai, Japan; 2-1 Seiryo-machi, Aoba-ku, Sendai, Miyagi 980-8575 Japan; 10 Department of Palliative Medicine, National Cancer Center Hospital, Tokyo, Japan; 5-1-1 Tsukiji, Chuo-ku, Tokyo, 104-0045 Japan

**Keywords:** Refractory cancer pain, Interventional procedures, Availability, Related factors, Nationwide survey

## Abstract

**Background:**

Cancer pain may be refractory to standard pharmacological treatment. Interventional procedures are important for quality of analgesia. The aim of the present study was to clarify the availability of four interventional procedures (celiac plexus neurolysis/splanchnic nerve neurolysis, phenol saddle block, epidural analgesia, and intrathecal analgesia), the number of procedures performed by specialists, and their associated factors. In addition, we aimed to establish how familiar home hospice physicians and oncologists are with the different interventional procedures available to manage cancer pain.

**Methods:**

A cross-sectional survey using a self-administered questionnaire was conducted. Subjects were certified pain specialists, interventional radiologists, home hospice physicians, and clinical oncologists.

**Results:**

The numbers of valid responses/mails were 545/1,112 for pain specialists, 554/1,087 for interventional radiology specialists, 144/308 for home hospice physicians, and 412/800 for oncologists. Among pain specialists, depending on intervention, 40.9-75.2% indicated that they perform each procedure by themselves, and 47.5-79.8% had not performed any of the procedures in the past 3 years. Pain specialists had performed the four procedures 4,591 times in the past 3 years. Among interventional radiology specialists, 18.1% indicated that they conduct celiac plexus neurolysis/splanchnic nerve neurolysis by themselves. Interventional radiology specialists had performed celiac plexus neurolysis/splanchnic nerve neurolysis 202 times in the past 3 years. Multivariate analysis revealed that the number of patients seen for cancer pain and the perceived difficulty in gaining experience correlated with the implementation of procedures among pain specialists. Among home hospice physicians and oncologists, depending on intervention, 3.5-27.1% responded that they were unfamiliar with each procedure.

**Conclusions:**

Although pain specialists responded that the implementation of each intervention was possible, the actual number of the interventions used was limited. As interventional procedures are well known, it is important to take measures to ensure that pain specialists and interventional radiology physicians are sufficiently utilized to manage refractory cancer pain.

## Background

Pain is a common symptom associated with cancer that needs to be controlled or reduced as much as possible. Cancer-related pain decreases the quality of life of patients [1, 2]. Pharmacological management is the basis of cancer pain treatment, and may adequately relieve cancer pain [3, 4]. However, a recent meta-analysis revealed that the proportion of patients with pain remains high, with 66.4% of patients with advanced terminal cancer having pain and 38% of those with cancer of any stage having moderate to severe pain [5].

The pharmacological management of cancer pain in some patients remains insufficient. Refractory cancer pain, which is defined as pain not responding to standard pharmacological treatments [6], may afflict some patients. The limitations of pharmacological therapy include its use for relief of breakthrough pain and side effects of analgesics. The use of individualized pharmacotherapy that considers the timing of treatment, individual characteristics, and non-pharmacological therapies is important for cancer-related pain. Among non-pharmacological therapies, the WHO guidelines [7] strongly recommend radiotherapy. Furthermore, authoritative guidelines [8–10] include non-pharmacological therapies such as neural blockade, neuraxial infusion, and cordotomy. Thus, in cancer pain management, an individualized multimodal approach is important [11, 12].

The degree to which interventional procedures for patients with cancer pain are available and utilized remains unclear. Some non-pharmacological therapies, including neural blockade and neuraxial infusion, are effective for cancer pain, and previous studies have reported that they are used to treat 3.8-8% of cancer patients [13–15]. However, as there are several barriers to the implementation of these therapies [16–21], their limited availability may explain refractory cancer-related pain in some patients with cancer.

Information on the status and availability of neural blockades and neuraxial infusions for cancer pain management or the factors associated with their use are currently limited [13–22]. Previous questionnaire surveys targeted palliative care physicians, referring physicians, and representatives of facilities at which treatment was provided [16–20, 23]; however, a national survey of the individual professionals who completed these surveys has not yet been performed.

The purpose of the present study was to clarify the availability and number performed by each specialist of four interventional procedures (celiac plexus neurolysis/splanchnic nerve neurolysis [CPN], subarachnoid neurolytic block for perineal pain [phenol saddle block], epidural infusions of local anesthetic combined with opioids [Epi], and intrathecal analgesia [IA] for refractory cancer pain) as well as factors related to their implementation using a nationwide survey of specialists. In addition, we aimed to clarify how familiar home hospice physicians (HHPs) and oncologists were with the different interventional procedures available to manage refractory cancer pain.

## Methods

### Study Design

A cross-sectional study on interventional procedures performed by pain specialists (PSs), interventional radiology (IVR) specialists, HHPs, and oncologists was conducted in Japan.

This survey was part of the “Research on the Construction of Systematic Pain Relief Methods in the Final Stage of Cancer Patients’ Medical Care” program.

### Participants and procedures

Between February and March 2020, a questionnaire on interventional procedures for refractory cancer pain was sent to PSs, IVR specialists, HHPs, and oncologists. Eligibility criteria were certificated physicians of each academic society. Exclusion criteria were: 1) not living in Japan, 2) not working at a hospital (regarding PSs and oncologists), and 3) no contact information. To identify subjects, we used lists of certified physicians from websites or certifying societies. A questionnaire was mailed to all certified PSs, IVR specialists, and HHPs who met the eligibility criteria, and to 800 oncologists randomly selected based on prefecture-based population ratios. Double board-certified oncologists who were certified as PSs or palliative care physicians were excluded from the analysis of valid responses.

A letter of purpose, questionnaire, and self-addressed envelope were enclosed and mailed, and a request was made in the letter of purpose to reply within one month of receipt of the questionnaire. A reminder by postcard was sent if when the questionnaire was not returned within this time.

### Measurements

In the present study, refractory pain was defined to participants as: pain that patients, family members, or nurses requested the physician to alleviate further, even with appropriate pharmacological therapy. All participants were asked about the following background factors: age, sex, the number of cancer patients seen annually, the number of cancer patients with pain seen annually, the number of cancer patients who died annually, other specialties, facilities at which they work, and their main workplace.

Self-administered questionnaires about the following interventional procedures for refractory cancer pain were conducted: CPN, phenol saddle block, Epi, and IA. We did not distinguish between celiac plexus neurolysis and splanchnic nerve neurolysis from the viewpoint of performing neural blockades for upper abdominal pain, even though the techniques and injection sites of neurolytic agents differ. PSs reported whether they currently perform these four therapies (yes/no), whether they are willing to perform them in the future (a four-point Likert scale consisting of “will perform”, “will probably perform”, “will probably not perform”, and “will never perform”), the number of procedures they performed in the past three years, and background factors and barriers related to the implementation of the four procedures. IVR specialists were asked about CPN only: whether they were currently performing CPN, whether they were willing to perform it in the future, and the number of procedures they had performed in the past 3 years.

Questions were based on those reported in previous studies [16–21, 23] and were developed through discussions among members of an expert group. The answers to potential barrier-related questions, such as experience, lack of time, communication with other departments, permission to perform at own facility, and availability of equipment at own facility, were recorded on a seven-point Likert scale with the following available responses: “strongly agree”, “agree”, “somewhat agree”, “neither agree nor disagree (undecided)”, “somewhat disagree”, “disagree”, and “strongly disagree”

Knowledge of these interventional procedures by HHPs and oncologists was also evaluated. HHPs and oncologists reported their knowledge and experience of interventional procedures for cancer pain management using one of four items: “I have performed the interventional procedure by myself”; “Some of my patients have received the interventional procedure from another physician”; “I know the interventional procedure, but have no experience with it”; and “I do not know the interventional procedure”.

### Analysis

Analyses were performed on valid responses using descriptive statistics. Responses regarding willingness to perform were divided into two categories: “will perform” and “will perform probably” were categorized as “willing”; and “will probably not perform” and “will never perform” as “not willing”. Responses expressed on a seven-point Likert scale were divided into two categories: “strongly agree” and “agree” were categorized as “agree”; and “somewhat agree”, “neither agree nor disagree (undecided)”, “somewhat disagree”, “disagree”, and “strongly disagree” as “other”. A univariate analysis of the factors and barriers that contribute to the implementation of interventional procedures was conducted using chi-squared test. Multivariate analysis (binomial logistic regression analysis) was performed on variables with a P value of ≤ 0.1 in the univariate analysis. P values < 0.05 were considered to be significant due to the exploratory nature of the present study. Items with missing values of 10% or more were excluded from the analysis. All analyses were conducted using SPSS (version 25, SPSS Inc., Chicago, USA) and R version 4.0.3.

## Results

### Response rate

**Fig. 1:**
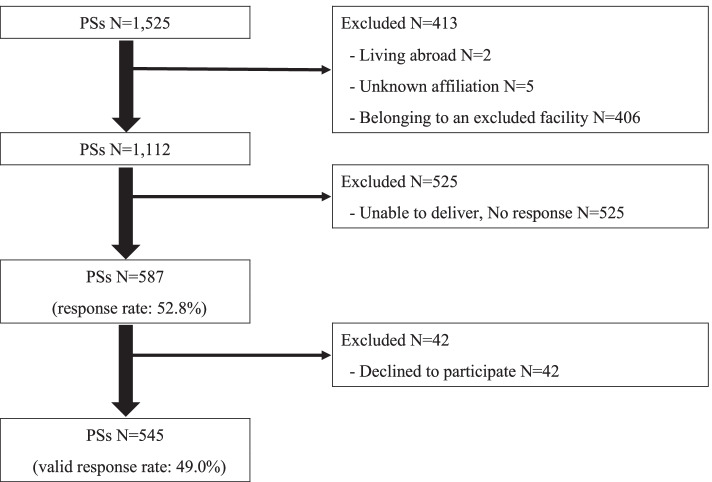
Participant flow (Pain specialist response rate). PSs: Pain specialists

**Fig. 2:**
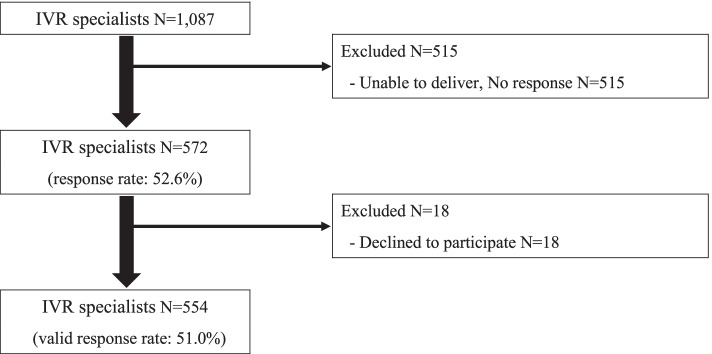
Participant flow (Interventional radiology specialist response rate). IVR: Interventional Radiology

Questionnaires were sent to 1,112 out of 1,525 PSs; 1,087 IVR specialists; 308 HHPs; and 800 randomly selected physicians out of 16,717 oncologists. Valid responses were obtained from 545/587 PSs (49.0%) (Fig. 1), 554/572 IVR specialists (51.0%) (Fig. 2), 144/146 HHPs (46.8%), and 399/425 randomly selected physicians (49.9%).

### Characteristics

Participant characteristics are shown in Table 1. The mean ages of PSs, IVR specialists, HHPs, and oncologists were 53.1, 48.2, 47.2, and 46.7 years, respectively. The median numbers of the four types of specialists who saw cancer patients with pain annually were 10, 3, 20, and 10, respectively. The proportions of the four types of specialists working in a designated cancer hospital or university hospital were 59.4, 67.5, 6.3, and 56.1%, respectively.

**Table 1:** Participant characteristics

	Pain specialists (*N=*545)	IVR specialists (*N=*554)	Home hospice physicians (*N=*144)	Oncologists (*N=*399)
Age, years mean±SD	53.1±9.3	48.2±9.5	47.2±9.2	46.7±7.7
Sex N (%)				
Male	372 (68.3)	510 (92.1)	104 (72.2)	333 (83.5)
Female	167 (30.6)	42 (7.6)	38 (26.4)	59 (14.8)
Cancer patients seen annually, median (IQR)	10 (2-100)	70 (20-200)	30 (15-50)	100 (35-200)
Cancer patients with pain seen annually, median (IQR)	10 (2-55)	3 (0-10)	20 (9.25-40)	10 (5-25)
Cancer patients who died annually, median (IQR)	3 (0-20)	-	20 (8.75-40)	10 (4-15)
Other specialties N (%)				
Internal medicine	12 (2.2)	22 (4.0)	65 (45.1)	85 (21.3)
Surgery	2 (0.4)	3 (0.5)	11 (7.6)	149 (37.3)
Anesthesiology	463 (85.0)	1 (0.2)	5 (3.5)	0 (0)
Family practice	7 (1.3)	3 (0.5)	46 (31.9)	2 (0.5)
Oncology	1 (0.2)	0 (0)	0 (0)	15 (3.8)
Radiology	1 (0.2)	503 (90.8)	1 (0.7)	20 (5.0)
Palliative medicine	112 (20.6)	0 (0)	17 (11.8)	5 (1.3)
Working facility N (%)				
Designated cancer hospital/university hospital	324 (59.4)	374 (67.5)	9 (6.3)	224 (56.1)
Other	221 (40.6)	180 (32.5)	135 (93.8)	175 (43.9)
Main workplace N (%)				
Ward/outpatient clinic	222 (40.7)	-	-	-
Operating room	294 (53.9)	-	-	-
Other	12 (2.2)	-	-	-

*IVR* Interventional radiology, *SD* Standard deviation, *IQR* Interquartile range

### Implementation of and preferences for interventional procedures

Table 2 shows the implementation of and preferences for interventional procedures. The proportions of PSs who indicated “Currently performing” and “Willing to perform in the future” for the various interventional procedures were as follows: CPN, 49.5 and 60.0%; phenol saddle block, 55.2 and 63.1%; Epi, 75.2 and 67.7%; and IA, 40.9 and 55.2%, respectively. Regarding the frequency of these procedures performed by PSs in the past 3 years, median numbers (interquartile ranges) for the various interventional procedures were as follows: CPN, 0 (0-3); phenol saddle block, 0 (0-1); Epi, 0 (0-3); and IA, 0 (0-0). The numbers of PSs who performed 20 or more procedures were 20 (3.7%), 4 (0.7%), 25 (4.6%), and 4 (0.8%), respectively.

**Table 2:** Implementation of and preferences for interventional procedures

	Pain specialists			IVR specialists		
	N	%	95% CI	N	%	95% CI
Celiac plexus neurolysis/splanchnic nerve neurolysis						
Currently performing						
yes	270	49.5	45.3-53.8	100	18.1	14.9-21.5
no	267	49.0	44.7-53.3	444	80.1	76.6-83.4
missing	8	1.5	-	10	1.8	-
Willing to perform in the future						
willing	327	60.0	55.8-64.1	278	50.2	45.9-54.4
not willing	207	38.0	33.9-42.2	260	46.9	42.7-51.2
missing	11	2.0	-	16	2.9	-
Number of implementations in the past 3 years						
Median (IQR)	0 (0-3)			0 (0-0)		
0	322	59.1		487	87.9	
1-4	125	22.9		35	6.3	
5-9	42	7.7		8	1.4	
10-19	27	5.0		6	1.1	
20-49	18	3.3		1	0.2	
≥50	2	0.4		0	0	
Subarachnoid neurolytic block for perineal pain (phenol saddle block)						
Currently performing					-	
yes	301	55.2	50.9-59.5			
no	238	43.7	39.5-48.0			
missing	6	1.1	-			
Willing to perform in the future					-	
willing	344	63.1	58.9-67.2			
not willing	193	35.4	31.4-39.6			
missing	8	1.5	-			
Number of implementations in the past 3 years						
Median (IQR)	0 (0-1)					
0	342	62.8			-	
1-4	150	27.5			-	
5-9	24	4.4			-	
10-19	16	2.9			-	
20-49	4	0.7			-	
≥50	0	0			-	
Epidural infusions of local anesthetic combined with opioids						
Currently performing					-	
yes	410	75.2	71.4-78.8			
no	107	19.6	16.4-23.2			
missing	28	5.1	-			
Willing to perform in the future					-	
willing	369	67.7	63.6-71.6			
not willing	151	27.7	24.0-31.7			
missing	25	4.6	-			
Number of implementations in the past 3 years						
Median (IQR)	0 (0-3)					
0	259	47.5			-	
1-4	144	26.4			-	
5-9	43	7.9			-	
10-19	45	8.3			-	
20-49	22	4.0			-	
≥50	3	0.6			-	
Intrathecal analgesia						
Currently performing					-	
yes	223	40.9	36.8-45.2			
no	321	58.9	54.6-63.1			
missing	1	0.2	-			
Willing to perform in the future					-	
willing	301	55.2	50.9-59.5			
not willing	240	44.0	39.8-48.3			
missing	4	0.7	-			
Number of implementations in the past 3 years						
Median (IQR)	0 (0-0)					
0	435	79.8			-	
1-4	81	14.9			-	
5-9	10	1.8			-	
10-19	9	1.7			-	
20-49	2	0.4			-	
≥50	2	0.4			-	

*IVR* Interventional radiology, *IQR* Interquartile range, *CI* Confidence interval

The proportions of IVR specialists who indicated “Currently performing CPN” and “Willing to perform CPN in the future” were 18.1 and 50.2%, respectively. Regarding the frequency of CPN performed by IVR specialists in the past 3 years, the median number (interquartile range) was 0 (0-0), with nearly 90% answering “0”.

In the past 3 years, 545 PSs reported performing 4,591 of the four procedures (CPN, 1547; phenol saddle block, 706; Epi, 1746; and IA, 592), whereas 554 IVR specialists reported performing 202 CPN.

### Factors related to the implementation of procedures by PSs

Univariate (Table 3) and multivariate analyses (Table 4) revealed that the number of cancer patients with pain seen annually and difficulty in gaining experience and acquiring skills due to the limited number of cases were associated with the implementation of all four interventional procedures for cancer pain management. Implementation not being permitted at the PSs’ own facility was a barrier to the implementation of CPN, phenol saddle block, and IA. The difficulty of treating patients requiring the procedure due to a lack of time was a barrier to the implementation of CPN and phenol saddle block. Items regarding equipment were excluded from the analysis because they were missing values of 10% or more.

**Table 3:** Factors related to the implementation of procedures by pain specialists (univariate analysis)

	Celiac plexus neurolysis/splanchnic nerve neurolysis			Phenol saddle block			Epidural infusions of local anesthetic combined with opioids			Intrathecal analgesia		
Variables	Currently implementing	Not currently implementing	*p*-value	Currently implementing	Not currently implementing	*p*-value	Currently implementing	Not currently implementing	*p*-value	Currently implementing	Not currently implementing	*p*-value
**Background**												
Age N (%)												
-39	19 (47.5)	21 (52.5)	0.526	24 (58.5)	17 (41.5)	0.933	33 (86.8)	5 (13.2)	0.426	17 (41.5)	24 (58.5)	0.915
40-59	173 (49.4)	177 (50.6)		196 (55.7)	156 (44.3)		269 (79.4)	70 (20.6)		143 (40.4)	211 (59.6)	
≥60	75 (54.7)	62 (45.3)		77 (56.6)	59 (43.4)		101 (77.1)	30 (22.9)		59 (42.4)	80 (57.6)	
Sex N (%)												
Male	196 (53.4)	171 (46.6)	0.043*	211 (57.5)	156 (42.5)	0.274	279 (79.5)	72 (20.5)	0.875	155 (41.8)	216 (58.2)	0.622
Female	72 (43.9)	92 (56.1)		87 (52.4)	79 (47.6)		127 (78.9)	34 (21.1)		66 (39.5)	101 (60.5)	
Number of cancer patients with pain treated annually N (%)												
0	14 (16.7)	70 (83.3)	<0.001*	18 (21.4)	66 (78.6)	<0.001*	42 (50.6)	41 (49.4)	<0.001*	14(16.7)	70 (83.3)	<0.001*
1-9	74 (44.8)	91 (55.2)		86 (52.4)	78 (47.6)		132 (84.1)	25 (15.9)		59(35.3)	108 (64.7)	
10-49	79 (64.2)	44 (35.8)		85 (68.5)	39 (31.5)		106 (87.6)	15 (12.4)		70(56.0)	55 (44.0)	
≥50	98 (67.6)	47 (32.4)		102 (69.4)	45 (30.6)		117 (84.8)	21 (15.2)		73(49.3)	75 (50.7)	
Working facility N (%)												
Designated cancer hospital/university hospital	176 (55.2)	143 (44.8)	0.006*	196 (60.9)	126 (39.1)	0.004*	249 (80.8)	59 (19.2)	0.294	137 (42.3)	187 (57.7)	0.457
Other	94 (43.1)	124 (56.9)		105 (48.4)	112 (51.6)		161 (77.0)	48 (23.0)		86 (39.1)	134 (60.9)	
Main workplace N (%)												
Ward/outpatient clinic	143 (65.0)	77 (35.0)	<0.001*	140 (63.3)	81 (36.7)	0.015*	170 (82.9)	35 (17.1)	0.016*	110 (49.5)	112 (50.5)	0.004*
Operating room	114 (39.3)	176 (60.7)		147 (50.7)	143 (49.3)		222 (77.9)	63 (22.1)		103 (35.0)	191 (65.0)	
Other	4 (36.4)	7 (63.6)		6 (50.0)	6 (50.0)		6 (50.0)	6 (50.0)		5 (41.7)	7 (58.3)	
**Barriers**												
Difficult to gain experience and acquire skills due to the limited number of cases N (%)												
Agree	109 (38.0)	178 (62.0)	<0.001*	68 (36.2)	120 (63.8)	<0.001*	17 (47.2)	19 (52.8)	<0.001*	43 (25.0)	129 (75.0)	<0.001*
Other	160 (65.6)	84 (34.4)		232 (67.2)	113 (32.8)		391 (82.1)	85 (17.9)		176 (48.6)	186 (51.4)	
Difficult to treat patients who require the procedure due to a lack of time N (%)												
Agree	39 (28.3)	99 (71.7)	<0.001*	25 (25.3)	74 (74.7)	<0.001*	38 (55.9)	30 (44.1)	<0.001*	22 (22.7)	75 (77.3)	<0.001*
Other	229 (58.4)	163 (41.6)		275 (63.2)	160 (36.8)		369 (83.5)	73 (16.5)		199 (45.4)	239 (54.6)	
Difficult to communicate with other departments when implementing the procedure N (%)												
Agree	27 (38.6)	43 (61.4)	<0.001*	17 (32.1)	36 (67.9)	<0.001*	22 (56.4)	17 (43.6)	<0.001*	18 (26.1)	51 (73.9)	0.006*
Other	242 (52.5)	219 (47.5)		284 (59.0)	197 (41.0)		385 (81.6)	87 (18.4)		203 (43.4)	265 (56.6)	
Implementation at our facility is not permitted N (%)												
Agree	6 (16.7)	30 (83.3)	<0.001*	7 (17.1)	34 (82.9)	<0.001*	7 (38.9)	11 (61.1)	<0.001*	3 (6.8)	41 (93.2)	<0.001*
Other	261 (53.3)	229 (46.7)		292 (49.5)	298 (50.5)		400 (81.1)	93 (18.9)		218 (44.3)	274 (55.7)	
Dispensing and using phenol glycerin are not permitted by the Ethics Committee or Regulatory Committee in our facility N (%)												
Agree	-	-		43 (40.2)	64 (59.8)	<0.001*	-	-		-	-	
Other	-	-		255 (60.3)	168 (39.7)		-	-		-	-	
The facilities to which patients may be referred after implementation are limited N (%)												
Agree	-	-		-	-		-	-		105 (39.6)	160 (60.4)	0.446
Other	-	-		-	-		-	-		117 (42.9)	156 (57.1)	

*significantly different

**Table 4:** Factors related to the implementation of procedures by pain specialists (multivariate analysis)

	Celiac plexus neurolysis/splanchnic nerve neurolysis			Phenol saddle block			Epidural infusions of local anesthetic combined with opioids			Intrathecal analgesia		
	OR	95% CI	*p*-value	OR	95% CI	*p*-value	OR	95% CI	*p*-value	OR	95% CI	*p*-value
**Background**												
Sex												
Male	REFERENCE		0.066	-		-	-		-	-		-
Female	0.658	0.42-1.03		-	-		-	-		-	-	
Number of cancer patients with pain treated annually												
0	REFERENCE		<0.001*	REFERENCE		<0.001*	REFERENCE		<0.001*	REFERENCE		<0.001*
1-9	3.72	1.84-7.51		4.14	2.15-7.97		4.94	2.54-9.60		2.74	1.38-5.44	
10-49	5.92	2.84-12.32		7.03	3.47-14.23		5.65	2.71-11.82		6.09	2.99-12.41	
≥50	5.77	2.68-12.42		8.02	3.80-16.92		5.13	2.38-11.08		4.11	1.98-8.52	
Working facility												
Designated cancer hospital/university hospital	REFERENCE		0.599	REFERENCE		0.04*	-		-	-		-
Other	1.12	0.73-1.74		1.58	1.02-2.43		-	-		-	-	
Main workplace												
Ward/outpatient clinic	REFERENCE		0.035*	REFERENCE		0.959	REFERENCE		0.199	REFERENCE		0.387
Operating room	0.57	0.36-0.90		1.05	0.65-1.70		1.12	0.62-2.02		0.77	0.50-1.20	
Other	0.37	0.09-1.49		1.18	0.31-4.49		0.34	0.09-1.28		1.51	0.35-6.49	
**Barriers**												
Difficult to gain experience and acquire skills due to the limited number of cases												
Agree	REFERENCE		<0.001*	REFERENCE		<0.001*	REFERENCE		0.006*	REFERENCE		<0.001*
Other	2.67	1.76-4.05		2.71	1.72-4.27		3.29	1.41-7.66		2.31	1.47-3.63	
Difficult to treat patients who require the procedure due to a lack of time												
Agree	REFERENCE		0.004*	REFERENCE		0.01*	REFERENCE		0.139	REFERENCE		0.318
Other	2.13	1.28-3.55		2.22	1.21-4.10		1.74	0.84-3.63		1.37	0.740-2.54	
Difficult to communicate with other departments when implementing the procedure												
Agree	REFERENCE		0.455	REFERENCE		0.956	REFERENCE		0.343	REFERENCE		0.96
Other	0.77	0.39-1.52		0.98	0.43-2.24		1.62	0.60-4.38		0.98	0.48-2.01	
Implementation at our facility is not permitted												
Agree	REFERENCE		0.009*	REFERENCE		0.002*	REFERENCE		0.193	REFERENCE		0.001*
Other	4.20	1.44-12.25		5.53	1.84-16.63		2.38	0.64-8.79		7.77	2.22-27.11	
Dispensing and using phenol glycerin are not permitted by the Ethics Committee or Regulatory Committee in our facility												
Agree	-	-		REFERENCE		0.402	-		-	-		-
Other	-	-		1.28	0.72-2.25		-	-		-	-	

*OR* Odds ratio, *CI* Confidence interval; * significantly different

### Perceptions of interventional procedures by HHPs and oncologists

The proportions of HHPs and oncologists who responded that they did not know each of the four interventional procedures were as follows: CPN, 7.6 and 13.0%; phenol saddle block, 13.9 and 19.0%; Epi, 3.5 and 6.5%; and IA, 11.1 and 27.1%, respectively (Table 5).

**Table 5:** Number of home hospice physicians and oncologists who responded that they did not know interventional procedures

	Home hospice physicians (*N=*144)			Oncologists (*N=*399)		
	N	%	95% CI	N	%	95% CI
Celiac plexus neurolysis/splanchnic nerve neurolysis	11	7.6	3.9-13.3	52	13.0	3.9-13.3
Subarachnoid neurolytic block for perineal pain (phenol saddle block)	20	13.9	8.7-20.6	76	19.0	15.3-23.3
Epidural infusions of local anesthetic combined with opioids	5	3.5	1.1-7.9	26	6.5	4.3-9.4
Intrathecal analgesia	16	11.1	6.5-17.4	108	27.1	22.8-31.7

*CI* Confidence interval

## Discussion

The present results clarified the availability, status of implementation, and factors related to the implementation of interventional procedures for refractory pain in patients with cancer using a nationwide survey completed by specialists.

In the present study, the proportions of PSs who responded that they were able to perform CPN, phenol saddle block, Epi, and IA were 49.5, 55.2, 75.2, and 40.9%, respectively. In the past three years, almost 50% reported that they had not performed Epi; furthermore, most responded that they had not performed the three other procedures. Previous surveys on specialist pain services examined the availability of interventional procedures. In the UK, procedures were available at 24.5% (CPN), 24.5% (intrathecal neurolysis), and 85.8% (spinal analgesia; 22% for EPI only, 18% for IA only, and 45% for both) of facilities [16]. In Japan, procedures were available at 66% (CPN), 67.4% (intrathecal neurolysis), 88.2% (Epi), and 54.2% (IA) of facilities [23]. Thus, many pain specialist facilities provide interventional analgesia for cancer patients; however, PSs had few opportunities to perform these procedures.

Based on a previous Japanese study [15], we estimated that 3.3% of the 373,584 patients who died due to cancer in 2018 (approximately 12,000 patients) may have required interventional procedures for cancer pain management. Our survey revealed that 1,530 interventional procedures were performed annually by 545 PSs. Assuming that the 1,112 PSs that responded to our survey performed interventional procedures at the same frequency as the 545 PSs, the expected annual number of interventional procedures was 3,122, which is markedly less than the estimated demand. Thus, interventional procedures do not appear to be sufficiently utilized.

Factors related to the implementation of interventional procedures warrant further study. Previous studies reported the following barriers to the implementation of specialist pain management, such as neural blockade and neuraxial infusion: the underutilization of specialists [16, 17]; access issues/geographical issues [18, 19]; inter-facility issues [19]; inability to get appointments [20]; need for repeating procedures [20]; cost issues [17, 18, 21]; the short survival of patients following referral to palliative care services [21]; time on the part of the specialist for evaluation and discussion [16, 21]; complexity [21]; continuity issues, such as the handling of pumps and catheters, creating a pump, procurement of drugs, and management at home [21]; the inexperience of palliative care physicians [18]; perception issues among palliative care physicians (interest or lack of awareness of potential benefits) [18, 21]; and the lack of training for specialists [21]. In the present study, the number of cancer patients with pain seen annually, difficulty in gaining experience, lack of time, and lack of institutional acceptance were associated with the implementation of procedures, with the first three factors being consistent with previously reported associated factors (involvement of specialists in palliative care [16], time on the part of the specialist for evaluation and discussion [16], and the lack of training for specialists [21]). These factors are important because the results of the present study support previous findings.

The following measures may increase the number of interventional procedures being performed. First, in the present survey, the number of cancer patients with pain seen annually (contributing factor) and difficulty in gaining experience and acquiring skills due to the limited number of cases (barrier) were identified as factors related to implementation. Moreover, previous studies reported the lack of training of experts as a barrier to implementation [21]. Thus, PSs need to increase their experience treating such patients. To increase the experience of PSs, several strategies may be effective, including further specialization for the treatment of cancer pain, a region-wide networking system for identifying potential candidates for interventional procedures, and establishing designated teaching facilities. Second, the effective use of time by PSs to practice palliative medicine may increase the implementation of procedures. In the present study, lack of time was associated with the implementation of two procedures: CPN and phenol saddle block. Moreover, increasing the time spent in palliative medicine practice may compensate for lack of experience. In a 2007 survey of lead anesthetists in UK pain clinics [16], joint consulting arrangements were rare, and only 25% of anesthetists’ job plans had time allocated for palliative medicine referrals; however, there was a positive correlation with the number of referrals. Therefore, promoting opportunities for PSs to be involved in palliative medicine may, in turn, increase the number of interventional procedures performed. Third, efforts are needed to educate palliative care physicians who will serve as bridges. The present survey of HHPs and oncologists revealed that they had knowledge of the implementation of procedures, but no experience or may not be able to refer patients to specialists. Palliative care physicians need to act as a bridge to connect patients to specialists who perform these procedures. Previous studies also reported a lack of experience and awareness among palliative care physicians [18, 21]; thus, further education and awareness on indications for and effects of interventional therapies among palliative care physicians are needed. Fourth, the education of IVR specialists may be important for promoting the implementation of CPN because even though many IVR specialists responded that they are willing to perform CPN, actual implementation rates were low.

Since there are few evidence-based interventional procedures, it may be difficult for specialists to provide a rationale for the procedure; furthermore, palliative care physicians who act as bridges may not be able to propose a procedure with confidence and obtain approval from institutions. Further studies to evaluate the efficacy of these interventional procedures are needed.

### Limitations

There are several limitations to the present study. First, although the status of implementation by specialists nationwide was surveyed, we did not obtain information on the implementation status of each facility. A survey of facilities, including designated cancer hospitals, hospitals without designated cancer departments, and home hospices is warranted to obtain more detailed data on interventional procedures for cancer pain management. Second, the valid response rate for each expert, which ranged between 46.8 and 51.5%, may not reflect the overall situation. However, the response rate was sufficient for a survey of individual experts. Third, as Japan has a universal health insurance system, no restrictions on access to medical facilities, and a small geographical area, we considered it unnecessary to ask about geographical distance and cost issues.

## Conclusion

PSs surveyed in the present study responded that they implement each of the four procedures to treat patients with cancer pain; however, the actual number appears to be limited and may not meet demands. It is important to take measures to ensure that PSs and IVR physicians are sufficiently utilized to manage refractory cancer pain.

## Data Availability

The datasets used and/or analyzed during the current study are available from the corresponding author on reasonable request.

## Abbreviations

CPN: Celiac plexus neurolysis/splanchnic nerve neurolysis
PSs: Pain specialists
IVR: Interventional radiology
HHPs: Home hospice physicians
Epi: Epidural infusions of local anesthetic combined with opioids
IA: Intrathecal analgesia
